# Epidemiological surveillance of tuberculosis-HIV co-infection and genotypic strains of the *Mycobacterium tuberculosis* complex in four departments at the *Centre Hospitalier Universitaire de Référence Nationale* in N’Djamena, Chad

**DOI:** 10.11604/pamj.2025.51.6.47259

**Published:** 2025-05-08

**Authors:** Gildas le Djimbaye Togde, Boilengar Djimenan, Djamalladine Mahamat Doungous, Abdelbassit Sahir Hassan, Gédéon Ossoga Walbang, Bassirou Diara, Bessimbaye Nadlaou, Rimtebaye Kimassoum

**Affiliations:** 1 Department of Biological and Pharmaceutical Sciences, Faculty of Human Health Sciences (FSSH), *Université de* N'Djamena, N'Djamena, Chad,; 2 Mycobacteria Unit, Service de Laboratoire, Centre Hospitalier Universitaire de Référence Nationale (CHU-RN), N'Djamena, Chad,; 3 Department of Biomedical and Pharmaceutical Sciences, *Institut National Supérieur des Sciences et Techniques d'Abéché*, BP 130 Abéché, Chad,; 4 United Medical Resources, Maladies Infectieuses et Vecteurs: Ecologie, Génétique, Evolution et Contrôle , Institut de Recherche pour le Développement-Centre National Recherche, Universitaire de Référence Nationale (UMR MIVEGEC, IRD-CNRS-UM-CHU), Montpellier, France,; 5 Department of Biology, *Ecole Normale Supérieure de* Bongor, Bongor, Chad,; 6 Centre Universitaire de Recherche Clinique (CURC), Université des Sciences, Techniques et Technologies de Bamako (USTTB), Point G, Bamako, Mali

**Keywords:** Epidemiological surveillance, TB/HIV coinfection, genotypic strains, Chad

## Abstract

**Introduction:**

the comorbidity of infectious diseases is a real public health challenge in Chad. The main objective of this study is to determine the prevalence rate of HIV/TB co-infection among patients of the Centre Hospitalier Universitaire de Référence Nationale (CHU-RN), as well as the frequency of circulating Mycobacterium tuberculosis complex (MTBc) genotypic strains within the institution.

**Methods:**

a cross-sectional analytical and prospective study was conducted from January to November 2024 in four departments of the CHU-RN in N'Djamena. Patient samples were collected, decontaminated, and analyzed using techniques such as the GeneXpert MTB/RIF test, MGIT culture, and spoligotyping.

**Results:**

one hundred and fifty-six (156) patients were included, with a majority of men at 65% and an average age of 38.28 years. Approximately 69.2% of the samples were positive for MTBc and the TB/HIV co-infection rate was 7.4%. The MTBc Cameroon strain was the most represented, with 43%, followed by the CAS-Delhi strain at 29%. Among the strains found at the CHU-RN, two lineages were revealed: L3 and L4. Rifampicin resistance of MTBc was 13%.

**Conclusion:**

the identification of MTBc genotypic strains shows high genetic diversity with a predominance of certain strains involved in co-infection with HIV at the CHU-RN. Targeted intervention is necessary to control the transmission of tuberculosis.

## Introduction

Tuberculosis (TB) remains an endemic disease in Chad with an estimated incidence of 134 cases per 100,000 population in 2023 [1]. Based on Chad´s total population, this translates to approximately 25,075 new cases [2]. The incidence is notably higher in men (69%) than in women (31%). Children accounted for 7.2% of recorded tuberculosis cases [2]. In 2023, the World Health Organization (WHO) reported 8.2 million new TB cases globally, the highest figure recorded since tuberculosis surveillance in 1995 [1]. However, the COVID-19 pandemic disrupted tuberculosis data collection in Africa, leading to a decline in reported cases from 7.1 million cases in 2019 to 5.8 million in 2020 [3,4]. It should be noted that recent developments in tuberculosis investigation have seen significant progress in genetic methods, notably IS6110-Restriction Fragment Length Polymorphism (RFLP) [5]. Several polymerase chain reaction (PCR) genotyping techniques (Spoligotyping, FluoroCycler® XT MTBDR, and MIRU determination) have been developed and continue to be discriminative and reproducible, with results at short intervals [1,5,6].

These PCR-based techniques can be used to demonstrate sensitivity to different anti-tuberculosis drugs and also to determine the different strains of the *M. tuberculosis* complex (MTBc) that may circulate within a community [5]. The MTBc is divided into nine lineages, whose genomic sequences are complete [7]. Lineage 1 is primarily found in India, where the number of tuberculosis cases is the highest in the world [8]. Lineage 2 originates from Asia but migrates to Europe and Africa. It is responsible for multidrug-resistant tuberculosis (MDR-TB) [7]. Strains from lineage 3 represent the main burden of tuberculosis, with high incidence in South Asia, North Africa, and East Africa [9]. Strains of this lineage carry potential MDR genes in certain parts of the world. In America, Europe, and Africa, lineage 4 (Euro-American) strains are the most widespread and are responsible for the high rate of tuberculosis/HIV co-infection [10]. Lineages 5/6 and 7 are found, respectively, in West Africa and Ethiopia. Recent work by Doungous *et al*. conducted in the *CHU-RN* during the COVID-19 pandemic period, found a rate of approximately 51.3% of *M. tuberculosis* circulating in this healthcare facility [11]. However, previous work carried out in 2012 by Ba Diallo *et al*., highlighted MDR-TB-carrying strains of MTBc distributed in certain regions of southern Chad [12]. Our study aims to determine different genotypic strains of MTBc that may sustain tuberculosis and the co-infection profile of patients in the *CHU-RN* to better understand aspects of TB surveillance.

## Methods

**Study design and framework:** we conducted an analytical and prospective cross-sectional study at the *CHU-RN* from January to November 2024. The analytic approach in our study seeks to examine the relationships between genotypic strains of *M. tuberculosis* and HIV/TB coinfection and treatment resistance. We collected samples in real time between January and November 2024 and data were recorded as cases emerged, enabling rigorous trend monitoring. Data collection in a single point provides a snapshot of surveillance at *CHU-RN* in 2024. This assesses the prevalence of TB-HIV co-infection and circulating strains. In our study, there are no experimental treatments or manipulations. Four departments were involved in the study: Pneumology-Phthisiology, Gastroenterology, Cardiology, and the Ear-Nose-Throat (ENT). The Mycobacteriology Laboratory at the *CHU-RN* in N'Djamena allowed us to decontaminate the samples and perform the Xpert MTB/RIF test. Then, our samples were sent to the Mycobacteriology and Hemorrhagic Fever laboratory at the University Clinical Research Center (UCRC) in Bamako for culture (BD, BACTEC MGIT 960) and Spoligotyping.

**Study population:** we included samples from new presumed TB patients and relapse patients admitted to one of the medical departments (Pneumology-Phthisiology, Gastroenterology, Cardiology and ENT) at *CHU-RN*. The age of the patients ranges from 15 years and older. We also considered both male and female patients. The age of the patients was taken as a criterion concerning the difficulty or poor quality of sputum production for patients under 15 years old.

**Sample collection, transport, and decontamination:** a rapid HIV test has been performed by clinicians before the samples of presumptive TB were sent to the laboratory. We received different types of samples at the Mycobacteria department based on their source departments: sputum and pleural fluid samples (pneumology-phthisiology), abdominal ascitic fluid samples (gastroenterology), pericardial fluid samples (cardiology) and lymph node pus samples (ENT). The samples were decontaminated using the Kubica method and then stored at -20°C [13]. We followed the International Air Transport Association (IATA) guidelines for biological products to send our samples to the UCRC laboratory in Bamako (Mali).

**GeneXpert MTB/RIF:** GeneXpert MTB/RIF is a computerized, automated molecular test for the detection of *Mycobacterium tuberculosis* complex (MTBc) and rifampicin susceptibility. Tests were performed according to the manufacturer's recommendations [14]. All samples received were analyzed by GeneXpert MTB/RIF.

**Culture and identification:** the samples were cultured on MGIT (Middle Growth Indicator Tube) medium in the Bactec 960 system. The MGIT detects mycobacterial colonies with an average incubation period of 7 to 14 days in the BACTEC 960 [15]. The sputum samples were digested and decontaminated using the standard N-Acetyl-L-Cysteine/4% NaOH solution, concentrated by centrifugation (3000 g), and inoculated on liquid media BD BBL MGITTM (Becton Dickinson and Compagny, Sparks, USA) and solid media (Middlebrook 7H11 Agar and Selective 7H11 Agar). Simultaneously, an aliquot of concentrated sediment was prepared for indirect Antimicrobial Resistance and Fluoroscein Diacetate (FDA). The speciation of positive mycobacterial cultures was based on Acid Fast Bacili positivity under the microscope and colony morphology on solid media, and it was confirmed by the Capilia TB-Neo assay (TAUNS Laboratories, Numazu, Japan).

**Spoligotyping:** culture-positive samples were used for spoligotyping. Using a commercially available kit (Ocimum) with an internally prepared membrane, we performed the spoligotyping technique using boiled bacterial lysates [16,17]. This technique amplifies the polymorphic region called DR (Direct Repeat) using two external primers (DRa and DRb) [18]. The PCR was carried out in real-time for amplification. Hybridization was then performed on an Isogen membrane. Development was done using a streptavidin conjugate and the Enhanced Chemi-Luminescence (ECL) detection kit (5). A filmstrip was used on the Isogen membrane in a darkroom for development and detection. The results were provided in octal code on the filmstrip, and strain comparison was performed with SPOTCLUST (based on SpolDB3) [5,19] and SITVIT2 [20]. This database is currently an important repository of genotypic markers, with over 111.635 genotypes isolated in 163 countries [20].

**Data and statistical analysis:** only tuberculosis patients with positive Xpert MTB/RIF results and culture-confirmed disease were included in the final analysis. Data were entered into Microsoft Excel, and statistical analysis was conducted using IBM SPSS version 2.5 software.

**Ethical considerations:** we ensured the confidentiality of patients´ clinical care. A research authorization from the ethics committee of the Human Health Faculty of Sciences has been delivered under N0309/CMT/PC/PMT/MESRI/SE/DGM/UNDJ/SG/SSH/21. Results have been returned to clinicians for the patients´ follow-up and to the National Tuberculosis Program for the epidemiological surveillance.

## Results

**Figure 1:**
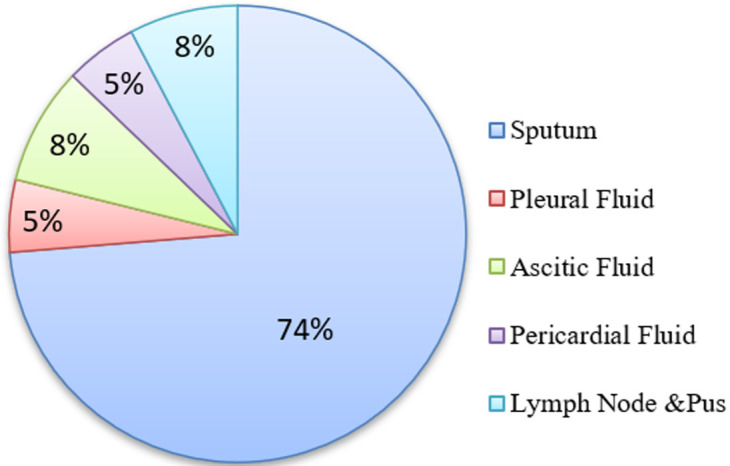
distribution of sample types received

We recorded a total of 156 patients from the four departments, of which 101 (65%) were men and 55 (35%) were women, with a sex ratio of 1.8 in favor of male patients. The average age of our patients was 38.28 years (ranging from 15 to 80 years). Four departments were identified in our study: pneumology-phthisiology, gastroenterology, cardiology, and ENT. We got different samples in the Mycobacterium department, sputum samples constituted the majority (74%) of our clinical samples (Figure 1).

**Overall prevalence of TB and HIV by sex at CHU-RN:** out of 156 recorded samples, 108 (69.2%) were MTBc positive in the Xpert MTB/RIF test. The male sex accounted for 69 (44.2%) MTBc positive samples, while the female sex accounted for 39 (25%). Table 1 shows the cases of TB detected by GeneXpert MTB/RIF and HIV distributed by department. The pneumonology-phthisiology department contained 56.4% of tuberculosis cases, followed by the ENT department with 7.1%. We found 8 (7.4%) cases of HIV positive among positive MTBc.

**Table 1:** detection of *Mycobacterium tuberculosis* complex and HIV status distributed by department

Departments N (%)	MTBc		HIV		Total
	Detected	Not-detected	HIV+	HIV-	
Pneumo-phtisiology	88 (56.4)	35 (22.4)	3 (1.9)	120 (77)	123
Gastro-enterology	7 (4.5)	6 (3.8)	4 (2.6)	9 (5.7)	13
Cardiology	2 (1.9)	6 (3.8)	0	8 (5.1)	8
ENT	11 (7.1)	1 (0.6)	1 (0.6)	11 (7.1)	12
Total	108 (69.2)	48	8 (5.1)	148	156

HIV: Human Immunodeficiency Virus; MTBc: *Mycobacterium tuberculosis* complex; ENT: Ears Nose and Throat

**Figure 2:**
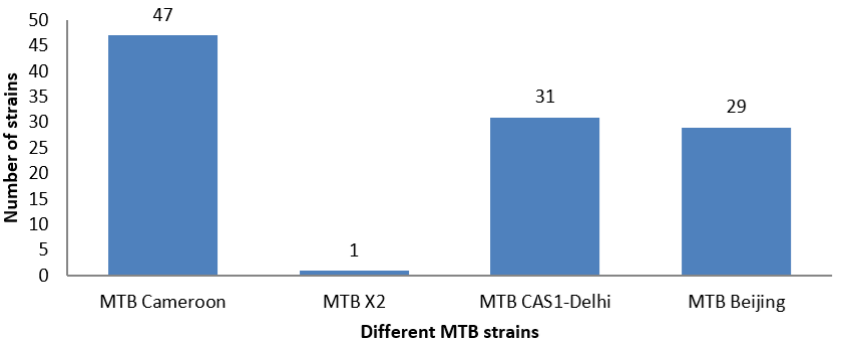
genotypic strains of *Mycobacterium tuberculosis* complex found at *CHU-RN*

**Different genotypic strains of *Mycobacterium tuberculosis* complex:** Figure 2 shows the frequency of different genotypic strains of the *Mycobacterium tuberculosis* complex. The MTB Cameroon strain is the most represented at CHU-RN with 43%, followed by the CAS1-Delhi strain (29%). Among the nine known lineages worldwide, two lineages, L3 and L4, have been detected. The L3 lineage includes MTB CAS1-Delhi, MTB Beijing, and MTB X2, while the L4 lineage contains the most widespread strain, MTB Cameroon.

**Isolated in the different departments and the rifampicin resistance in CHU-RN:** Table 2 presents the distribution of different genotypic strains and rifampicin resistance by department. Out of 108 tuberculosis patients, 14 (13%) are resistant to rifampicin. We did not find any relationship between the lineages and TB-R (OR 1.76 (95% CI 0.78-3.98)). Moreover, we found that male sex and seropositivity were associated with RR (OR 1.8 (95% CI 1.7-2,0)), and (OR 6.04 (95% CI 1.37-26.65)), respectively.

**Table 2:** distribution of isolated strains in different departments and the rifampicin resistance

Isolated strains (MTBc+)		Pneumo-phtisiology N (%)	Gastroenterology N (%)	Cardiology N (%)	ENT N (%)	Cumul N (%)
**Overall frequency/Department**		69 (44.2)	20 (12.8)	6 (3.8)	13 (8.3)	108 (69.2)
**Strains**	MTB Cameroon	26 (24)	5 (4.6)	3 (2.8)	13 (12)	47
	MTB CAS1-Delhi	23 (21.3)	1 (0.9)	2 (1,9)	5 (3.7)	31
	MTB Beijing	19 (17.6)	7 (6.5)	0	3 (2.8)	29
	MTB X2	0	0	0	1 (0.9)	1
**RIF resistant**		9	0	2	3	14 (13**)**

RIF:Rifampicin; MTBc: *Mycobacterium tuberculosis* complex; ENT: Ears Nose and Throat

**Association between the HIV status, TB status and age group of patients:** Table 3 shows the different age groups most affected by TB and associated HIV. New cases are higher than relapse cases, with approximately 18% of cases in the (15-25) age group. Additionally, relapse cases are more coinfected in the 956-65) age group.

**Table 3:** distribution by age group, and tuberculosis status of patients

TB-HIV status/age-group	HIV negative		HIV positive		Total	
	New cases-TB	Relapse-TB	Relapse-TB	New cases-TB	N	%
(15-25 )	33	6	2	3	44	28.2
(26-35)	19	15	1	0	35	22.4
(36-45)	17	13		1	32	20.5
(46-55)	18	9	0	1	28	17.9
(56-65)	9	3	0	0	12	7.7
(66 and over)	2	3	0	0	5	3.2
**Total**	98	49	3	5	156	

TB:Tuberculosis, HIV:Human Immunodeficiency Virus

## Discussion

We conducted an observational and prospective cross-sectional study at CHU-RN in N'Djamena from January to November 2024. We showed that two of the nine known genotypic lineages circulate within the National Reference University Hospital Center. This is relatively diverse but not as much as the West African border region, which is identified only by the presence of two other lineages of *M. africanum*, L5 and L6, according to Togo *et al*. 2018 [21]. Additionally, this work confirms the findings of Togde *et al.*, 2024, which identified three MTBC lineages circulating in six southern regions of Chad [22]. The MTB Cameroon strain is predominant at CHU-RN. This strain was first discovered in the Republic of Cameroon by Niobe *et al*. [23]. Previously identified in Chad by the work of Togde *et al*., 2024, in six southern regions, as well as by Ba Diallo *et al*. (2017) [12,22]. Given that the Republic of Cameroon shares a large border with Chad, controlling population movements at the border in response to economic and political pressures is challenging. This similarity in identified strains may suggest relative stability and endemicity of disease transmission in the CHU-RN. This hospital institution is the biggest in Chad, and it welcomes many foreigners. this cosmopolitan aspect might explain the origin of these strains that we found. One of the clinical utilities of spoligotyping is its rapid bacterial identification and comparison of strain molecular profiles, which is very useful in monitoring tuberculosis transmission and preventing disease spread.

The Pneumology-Phthisiology and ENT departments have a high rate of MTBc genotypic strains. This can be explained by the fact that the Pneumology-Phthisiology department receives all patients with pulmonary problems, and the ENT department receives all patients from various origins. The fact that all the human organs such nose and throat are linked directly to the lungs. Using Xpert MTB/RIF, we found a frequency of 13% TB-RR. We did not find any association between the lineages and TB-R (OR=1.76, 95% CI: [0.78-3.98]). In Ethiopia, Mulu *et al*., 2017 found a rifampicin resistance of 10% [24]. Comparing these findings with ours, we can say that the prevalence of TB-RR is higher in our study. This requires particular attention in monitoring resistance to anti-tuberculosis drugs. Our study has some limitations, notably the absence of complete tests for all included patients, such as comprehensive Drug Susceptibility Test for first and second-line drugs. Additionally, the number of patients included in this study cannot be extrapolated to the entire country, given that 25,000 new tuberculosis patients were detected in Chad in 2022 [25]. Finally, we only used spoligotyping to determine the strains, which could have been confirmed by another method, such as MIRU VNTR, and/or RFLP, or whole-genome sequencing. The data could be used to predict tuberculosis transmission and propose an effective public health response in the future. The epidemiological surveillance is urgent according to our findings.

## Conclusion

The molecular identification of genotypic strains of the *M. tuberculosis* complex at CHU-RN has shown relatively high diversity over the past six years. *M. tuberculosis* Cameroon within the modern Euro-American lineage was the most widespread. The continued predominance of these strains within the *M. tuberculosis* complex may suggest a stable and conserved host-pathogen interaction. Although the sample size is relatively small, targeted intervention is necessary to overcome this endemic disease in the region. Special attention should be given to the Pneumonology-Phthisiology department of the hospital to review the management of tuberculosis patients. With a rate of over 50% of MTBc detected in this department, we believe the team should be strengthened to achieve its goals.

### What is known about this topic

- There is a previous study in CHU-RN that gave information on the prevalence of tuberculosis in this institution after COVID-19;
- Other studies found Mycobacterium strains in the southern towns in Chad;
- Mycobacterium tuberculosis Cameroon is the most found strain in the whole country.

### What this study adds

- The study provides the diagnosis of tuberculosis with other biological fluids than sputum;
- This study detected Mycobacterium genotypic strains in four departments within the hospital institution;
- Our study confirms that the most frequent Mycobacterium strain is the Mycobacterium tuberculosis Cameroon.
